# Valorization of Prickly Pear Juice Geographical Origin Based on Mineral and Volatile Compound Contents Using LDA

**DOI:** 10.3390/foods8040123

**Published:** 2019-04-15

**Authors:** Vassilios K. Karabagias, Ioannis K. Karabagias, Artemis Louppis, Anastasia Badeka, Michael G. Kontominas, Chara Papastephanou

**Affiliations:** 1 Laboratory of Food Chemistry Department of Chemistry University of Ioannina, 45110 Ioannina, Greece; vkarambagias@gmail.com (V.K.K.); abadeka@uoi.gr (A.B.); mkontomi@cc.uoi.gr (M.G.K.); 2 cp Foodlab Ltd, Polifonti 25, Strovolos, Nicosia 2047, Cyprus; artemislouppis@gmail.com (A.L.); foodlab@cytanet.com.cy (C.P.)

**Keywords:** prickly pear, juice, minerals, aroma, authentication, chemometrics

## Abstract

In the present work the mineral content and volatile profile of prickly pear juice prepared from wild cultivars was investigated. Fruits used in the study originated from three areas of the Peloponnese Peninsula. Twenty-five macro- and micro-minerals (K, Na, P, Ca, Mg, Al, B, Ba, Be, Co, Cr, Cu, Fe, Li, Mn, Mo, Ni, Sb, Se, Si, Sn, Ti, Tl, V, Zn) were determined using inductively coupled plasma atomic emission spectroscopy (ICP-OES). Furthermore, analysis of the mineral content of soil samples with ICP-OES showed a perfect correlation with those of fruit juices. Volatile compounds (alcohols, aldehydes, hydrocarbons, terpenoids, and others) were identified using an optimized headspace solid phase microextraction coupled to gas chromatography mass spectrometry (HS-SPME/GC-MS) method. Multivariate analysis showed significant differences (*p* < 0.05) among the investigated parameters with respect to juice geographical origin. Prickly pear juice samples were classified according to geographical origin by 85.7% and 88.9% using 7 minerals and 21 volatile compounds, respectively.

## 1. Introduction

*Opuntia ficus indica* or the Indian fig opuntia is a fruit that belongs to the cactus family, Cactaceae [1]. The fruit originated from North and South America; however, it was also introduced in Europe and grows in regions with a suitable climate, such as the south of France, southern Italy (Sardinia, Sicily, etc.), Bulgaria, southern Portugal (Madeira), Spain (Andalusia), Albania, Cyprus, and Greece. Some typical regions in Greece where the fruit grows well are Peloponnese, the Ionian Islands, and Crete.

From a nutritional point of view, prickly pear fruit (portions of 100g) provides the human body with 41 calories and is composed of water (ca. 88%), carbohydrates (ca. 10%), and smaller amounts of fat, protein, vitamin C, and minerals [2]. Considerable amounts of phytochemicals (betalain, betanin, indicaxanthin, gallic acid, vanillic acid, catechins, etc.) have also been reported [3,4]. The fruit has a long term history in the Mexican culture as a natural healer of wounds and inflammation of the digestive and urinary tracts [5].

Fruit juices may comprise a delicious beverage with a characteristic flavor and taste, and may be a health companion in daily diets, due to the documented health benefits upon regular consumption. Some typical components that are responsible for the beneficial health effects of fruit juices include polyphenols, carotenoids, vitamins, minerals and trace elements [6,7]. Yet, a unique aroma may be the primary criterion for acceptance of a product among consumers. In that sense, when sensory and nutritional characteristics are well combined, these attributes may result in the development of a new product.

Some previous studies in the literature deal with the characterization of apple, orange, pear, peach, apricot, blueberry, cranberry, plum, lemon, and cherry juices by means of volatile compounds, polyphenols, spectra profiling, mineral and trace element analysis, and sensory metrics determination, using solid phase micro-extraction coupled to gas chromatography mass spectrometry (SPME-GC/MS), high performance liquid chromatography (HPLC), Fourier transform infrared spectroscopy (FTIR), nuclear magnetic resonance (1H-NMR), inductively coupled plasma atomic (or optical) emission spectrometry (ICP-AES or ICP-OES), inductively coupled plasma mass spectrometry (ICP-MS), instrumental neutronic activation analysis (INAA) and the electronic tongue [8,9,10,11,12,13,14].

As a matter of fact, application of innovative statistical approaches on the aforementioned chemical or sensory markers, namely principal component analysis (PCA), linear discriminant analysis (LDA), soft independent modeling of class analogies (SIMCA), hierarchical cluster analysis (HCA), k-nearest neighbor analysis (KNN), and partial least square-discriminant analysis (PLS-DA), has led to the development of accurate models for fruit juice authentication [7].

However, data involving the mineral content and volatile profile of prickly pear juice prepared from Greek wild-grown prickly pear fruits has not been previously reported. In addition, correlation of the mineral content of soil samples with those of fruit juice samples has been scarcely reported.

Considering the above, the objectives of the present study were to: (i) investigate the mineral content of prickly pear juice and correlate it with the mineral content of soil, where fruits of the naturally grown wild cultivars were collected, (ii) determine the volatile compounds that are responsible for prickly pear juice aroma, and (iii) check whether the differences in mineral or volatile compound content may be used for prickly pear juice differentiation according to geographical origin using a supervised chemometric tool, such as linear discriminant analysis. Practical applications of the present study may be focused on two basic axes: (i) exploitation of prickly pear juice and (ii) creation of a data base of mineral and volatile compound content of prickly pear juice prepared from wild fruit cultivars, as these are the intermediates for the cultivation of hybrid cultivars often termed as “less wild ones”.

## 2. Materials and Methods 

### 2.1. Prickly Pear Juice Samples

Thirty-six batch juice samples were prepared by machine squeezing (Rohnson fruit squeezer, power of 1000 W) of ca. 60 kg of prickly pear fruit naturally grown (wild cultivar) in the regions of East Messinia (12 samples), West Messinia (12 samples), and Lakonia (12 samples). This procedure (batch sampling) was followed in order to: (i) eliminate any existing differences in the maturity of the fruit used since these originated from different cactus plants and (ii) reduce the experimental cost of the study. Prior to juice preparation, fruit were tentatively washed with tap water, dried, and manually peeled. Samples were stored in polyethylene terephthalate (PET) containers (volume of 500 mL) and maintained at −18 ± 1 °C until analyses.

### 2.2. Chemicals and Multi-Element Standard

The chemicals used in the study were of analytical grade. The standard solutions of each mineral were prepared by appropriate dilution with ultrapure water (Milli-Q, Millipore, Bedford, MA, USA), of a multi-element standard (100 mg/L) obtained from Merck (Darmstadt, Germany). Nitric acid suprapure 65%, used for the digestion of samples, was obtained from Merck (Darmstadt, Germany) [15].

### 2.3. Determination of Mineral Content in Soil Samples

The mineral content of soil samples was determined according to the method of the Association of Official Analytical Chemists (AOAC) [16], which refers to the determination of metals in solid wastes, by using an inductively coupled plasma atomic emission spectrometric method (ICP-OES). The analysis of each sample was carried out in triplicate (*n* = 3). Results were expressed as mg/L.

### 2.4. Determination of Mineral Content in Prickly Pear Juice Samples

Approximately 0.5 g of each juice sample or fruit were weighed in microwave cup, previously rinsed with a mixture of superoxide and water (1:1). The sample was then mineralized using 7 mL 65% HNO_3_ (suprapure) and 2 mL of H_2_O_2_ 20% (*v*/*v*) (Merck, Darmstadt, Germany). The digestion was accomplished by heating the mixture for 10 and 20 min at 200 °C, respectively, using a microwave digester (power of 1000 W). The obtained mixture was then sonicated and diluted to a final volume of 100 mL with ultrapure water before ICP-OES analysis. The analysis of each sample was carried out in triplicate (*n* = 3). Results were expressed as mg/kg.

### 2.5. ICP-OES Instrumentation and Method Analytical Characteristics

A Thermo Scientific IRIS Intrepid II XDL inductively coupled plasma-atomic emission spectrometer (Thermo Electron Corporation, Waltham, MA, USA) was used for the elemental analysis. The emission wavelength (nm) was: 309.3, 455.4, 455.4, 313.0, 393.3, 228.6, 267.7, 324.7, 259.9, 766.5, 670.8, 279.5, 257.6, 202.0, 589.0, 221.6, 178.3, 206.8, 196.0, 251.6, 190.0, 334.9, 190.8, 292.4, 213.9, for Al, Ba, B, Be, Ca, Co, Cr, Cu, Fe, K, Li, Mg, Mn, Mo, Na, Ni, P, Sb, Se, Si, Sn, Ti, Tl, V and Zn, respectively. The operational parameters for the instrument were in accordance with a previous work [17]. For the determination of each amount of mineral, calibration curves were prepared. These showed correlation coefficients (*R*^2^) in the range of 0.9967–1.000 for soil and 0.997–1.000 for fruit juice samples, respectively (Supplementary Tables S1 and S2). Furthermore, the analytical method developed in the present study showed a satisfactory percent recovery for both soil and prickly pear juice samples at different spiking concentrations (six replicates) (Supplementary Tables S3 and S4). The limit of detection (LOD) and limit of quantification (LOQ) were estimated by spiking a blank sample (ultrapure water) three times with the standard mineral solution at low concentrations and the signal-to-noise ratio was determined. The LOD was defined as 3:1 and the LOQ as 10:1. The LOD and LOQ values for each mineral determined in soil and fruit juice samples are provided in the supplementary material (Supplementary Tables S5 and S6). Finally, the coefficient of variation for all minerals determined in prickly pear juice samples (*n* = 36) was ≤5.10% (Supplementary Table S7).

### 2.6. HS-SPME/GC-MS Analysis

#### 2.6.1. Method Optimization 

An optimization procedure was followed, in a preliminary experiment, in order to determine the most appropriate parameters for the extraction of volatile compounds from the headspace of prickly pear juice. These included: sample volume (5 and 10 mL), equilibrium time (10, 20, 25, and 30 min), sampling time (10 and 20 min), sample volume, extraction temperature (40, 42, and 45 °C), and salt (0, 20, and 30% *w*/*v*) addition [18,19]. Based on (i) the number of volatiles determined, (ii) the MS qualification results, (iii) the limited furan derivatives identified, and (iv) the spectra intensity along with the agreement in volatiles identified during the analysis of replicates, the optimum analysis conditions were found to be: 25 min equilibration time, 20 min sampling time, 5 mL sample volume, addition of salt (30% *w*/*v*), and 42 °C water bath temperature (Supplementary Table S8 and typical chromatograms (Figures S1–S8)).

#### 2.6.2. Extraction of Volatile Compounds

The volatile compounds of prickly pear juice were extracted using a divinyl benzene/carboxen/polydimethylsiloxane (DVB/CAR/PDMS) fiber 50/30 μm (Supelco, Bellefonte, PA, USA). The fiber was firstly conditioned, prior to use, according to the manufacturer’s recommendations. Afterwards, the samples consisting of 5 mL of juice, 1.5 g NaCl (Merck, Darmstadt, Germany), and 50 μL of internal standard (benzophenone, 100 μg/mL, Sigma Aldrich, St. Louis, MO, USA), were placed in 20 mL screw-cap vials equipped with polytetrafluoroethylene (PTFE) septa. The vials were maintained at 42 °C in a water bath under continuous stirring at 800 rpm during the headspace extraction. The stirring procedure usually improves the extraction efficiency [19]. For this purpose, a magnetic stirrer (cross shaped and coated with PTFE) with a diameter of 10 mm (Semadeni, Ostermundigen–Bern, Switzerland) was placed inside the vials. Fruit juice samples were prepared daily prior the HS-SPME-GC/MS analysis and the fiber was cleaned, before each sample analysis, using the clean program method. The analysis of each sample was run in duplicate and results were averaged.

#### 2.6.3. GC/MS Instrumentation and Analysis Conditions 

Volatile compound analysis of prickly pear juice samples was carried out using an Agilent gas chromatograph (Agilent 7890A) equipped with an Agilent mass detector (Agilent 5975). The chromatographic separation was achieved using DB-5MS (cross linked 5% PH ME siloxane) capillary column (60 m × 320 μm i.d., × 1 μm film thickness). Helium was used as the carrier gas (purity of 99.999%) at a flow rate of 1.5 mL/min. The injector and MS-transfer line were maintained at 260 °C and 280 °C, respectively. For HS-SPME analysis, the initial oven temperature of 40 °C was increased to 168 °C at a rate of 4 °C/min (0 min hold), and finally increased at a rate of 10 °C/min to 260 °C (1 min hold). The acquisition mode was scan. Electron impact mass spectra were recorded in the 29–350 mass range. An electron ionization system was used with an ionization energy of 70 eV. Solvent delay was set at 5 min to avoid co-elution of ethanol, since the internal standard was dissolved in ethanol [19]. Finally, a split ratio of 2:1 was used in the analysis. 

#### 2.6.4. Identification of Volatile Compounds

The Wiley 7, NIST 2005 mass spectral library was used for the identification of volatile compounds of prickly pear juice. The linear retention indices were also calculated for each compound, using a mixture of n-alkanes (C8–C20) dissolved in n-hexane. The standard mixture of alkanes was purchased by Fluka (Leipzig, Germany). The calculation was carried out for components eluting between n-octane and n-eicosane. Only the volatile compounds that had ˃85% similarity with Wiley library were tentatively identified using the GC-MS spectra. Results were expressed as a concentration (C_analyte_, μg/L) based on the peak area ratio of the isolated volatile compounds to that of the internal standard assuming a response factor equal to one for all the isolated compounds [18]. The internal standard (m/z = 182) used did not cause any co-elution problems, as it eluted as the final organic compound with no derivatives were identified.

### 2.7. Statistical Treatment of Data

Statistical treatment of data was performed using the SPSS 20.0 statistics software (IBM, Armonk, NY, USA). Comparison of average values (mineral content and semi-quantitative data of volatile compounds) was carried out based on a multivariate analysis of variance (MANOVA). MANOVA defined the minerals, or volatiles that were significant (*p* < 0.05) for the differentiation of prickly pear juice samples according to geographical origin. The Pillai’s trace and Wilks’ lambda indices were computed to determine a possible significant effect of minerals, or volatile compounds on the geographical origin of prickly pear fruit juice samples. Linear discriminant analysis (LDA) was then applied only to the selected independent variables by MANOVA, to explore the possibility of differentiating prickly pear juice samples according to geographical origin. For the LDA analysis geographical origin was taken as the dependent variable (grouping variable), while minerals or volatiles were taken as the independent variables [18,20].

More specifically, discriminant analysis creates a predictive model for group membership. The model is composed of one or more discriminant functions based on linear combinations of the predictor variables that provide the best discrimination between the constructed groups. The functions are generated from a sample of cases of known group membership. In addition, the functions can then be applied to the new cases that have measurements for the predictor variables but do not belong in a given group (unknown group membership). In that sense, predicted group membership may be well defined [21]. In addition, regarding the robustness of LDA analysis, Huberty and Olejnik [22] reported studies with a minimum of 10 observations per group. At the same time, what is also important is that the basic criteria (regularity, stability of variations, and independency of variations) should be met. Hence, in the present study these criteria were applied.

Furthermore, a tolerance test was also considered in the analysis. Tolerance is the proportion of a variable’s variance not accounted for by other independent variables in the discriminant function developed. A variable with very low tolerance contributes little information to a predictive model and may cause computational problems [23]. Finally, to provide correlations between mineral content of soil samples with those of prickly pear juices samples, Pearson’s correlation coefficient (*r*) was applied at the confidence level *p* < 0.05.

## 3. Results and Discussion

### 3.1. Mineral Content Analysis of Soil Samples

During the analysis, significant differences (*p* < 0.05) were observed in total and individual mineral content of soil samples with respect to geographical origin. Full data (average values, mg/L) are given in the supplementary material (Supplementary Table S9). The richest soil in minerals (sum of all minerals, mg/L) was that of the Lakonia region (102,756.15 mg/L), followed by the Eastern (82,246.76 mg/L) and Western Messinia (41,221.04 mg/L) regions, respectively. In addition, the presence of some chemical elements such as Cr, Ti, and Tl in soil samples recorded variations. In particular, Cr was identified in all soil samples, whereas Ti was identified only in soil samples from the region of Lakonia. Finally, Tl was not identified in any of the soil samples analyze. These findings indicate the unique/and or characteristic soil conditions in the soil samples investigated.

### 3.2. Mineral Content Analysis of Prickly Pear Juice Samples

As in the case of soil samples, the respective mineral content of prickly pear juice samples varied significantly (*p* < 0.05) according to geographical origin. The dominant minerals (mg/kg) were K, P, Ca, Mg, and Na, followed by B, Mn Zn, Sn, and Si. Total mineral content of prickly pear juice samples (average values, mg/kg) was obtained from the sum of each individual mineral and followed the order: Lakonia (2827.98 ± 310.65 mg/kg) > Eastern Messinia (2602.66 ± 203.51 mg/kg) > Western Messinia (2206.07 ± 214.77 mg/kg) (Table 1).

Trace minerals such as Be, Co, Mo, Sb, and Ti were not identified, whereas Cr was identified in two samples (no. 9 and 11 from Lakonia) at 0.03 mg/kg. The same holds for Tl, which was identified in two samples (no. 2) from Western Messinia and Lakonia at 0.04 and 0.20 mg/kg, respectively. Even though considerable amounts of Cr and Ti were identified in the soil samples analyzed (Supplementary Table S9), these elements were identified in minor amounts or were completely absent in the fruit juice samples analyzed. This is probably owed to a specific mineral accumulation mechanism that exists in prickly pear fruit of wild cultivars. On the other hand, despite the fact that Tl was not identified in the soil samples analyzed, this element was identified in prickly pear juice samples (no. 2) from Western Messinia and Lakonia at 0.04 and 0.20 mg/kg, respectively. This “paradox” may be justified by the fact that Tl exists in two oxidation states (+3) and (+1), as ionic salts. The +1 state is more prominent in Tl than the elements above it. Thallium somehow recalls the chemistry of alkali metals. In that sense, thallium (1) ions are found geologically in potassium-based ores, transmitted then to specific fruits, and are probably released through the preparation of prickly pear juice.

Finally, Ba recorded non-significant differences among the different geographical origins, ranging from 0.03–0.05 mg/kg (average value). Full data regarding the mineral content of minerals determined are given in the supplementary material (Supplementary Table S7). Dehbi et al. [24] reported higher contents of Ca, Mg, P, and Na in prickly pear juice prepared from Moroccan prickly pear cultivars, whereas those of K, Zn, and Cu were lower compared to results of the present study. Higher contents of Ca, K, P, Fe, and Mg were also reported by Mohamed et al. [25] involving Algerian prickly pear juice. However, the respective Na content was much lower compared to present results. The impact of geographical origin is of great importance to realize the differences between previous, even though limited, studies in the literature and results of the present study. Finally, there was also a perfect Pearson’s correlation (*r* = 1) (*p* < 0.05) between total mineral content of soil samples with those of prickly pear juice samples. 

### 3.3. Volatile Profile of Prickly Pear Juice 

**Figure 1:**
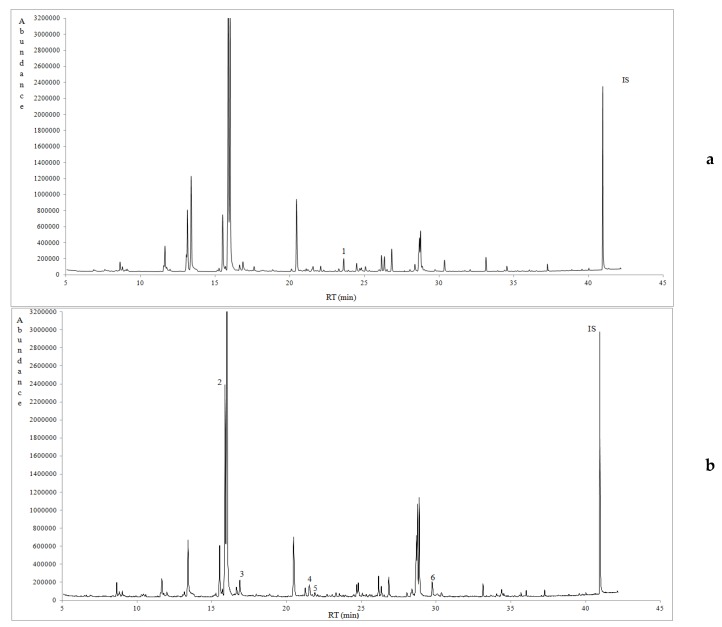

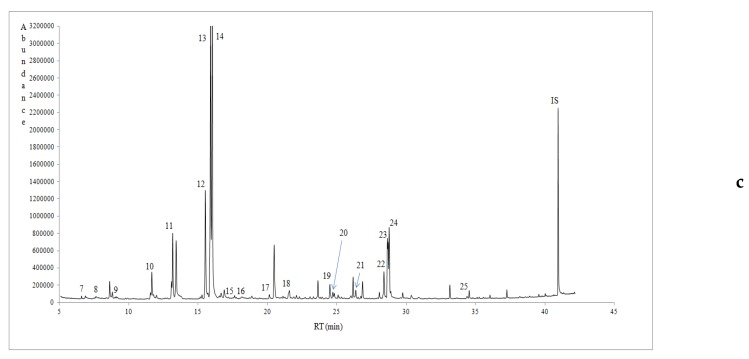
(**a**) A typical gas chromatogram of prickly pear juice sample (no. 12) from Western Messinia. 1: dl-Limonene. IS: internal standard. (**b**) A typical gas chromatogram of prickly pear juice sample (no. 3) from Eastern Messinia. 2: Hexanol. 3: 3,5-Hexadien-1-ol. 4: Decene. 5: Decane. 6: Dodecene. IS: Internal standard. (**c**) A typical gas chromatogram of prickly pear juice sample (no. 11) from Lakonia. 7: 2,4-Hexadiene. 8: 2-Butenal. 9: Pentanal. 10: Pentanol. 11: Hexanal. 12: 3-Hexen-1-ol. 13: 2-Hexenal. 14: 2-Hexen-1-ol. 15: Heptanal. 16: 2,4-Hexadienal. 17: 2-Heptenal. 18: 2-pentyl-Furan. 19: 2-Octenal. 20: Octanol. 21: Nonanal. 22: 2,6-Nonadienal. 23: 2,6-Nonadien-1-ol. 24: 2-Nonen-1-ol. 25: 2,4-Decadienal. IS: Internal standard.

The volatile pattern of prickly pear juice was dominated by alcohols and aldehydes, followed by lower amounts of hydrocarbons and specific terpenoids, such as dl-limonene, along with limited furan derivatives like 2-pentyl-furan. In total, 25 volatile compounds were tentatively identified and then semi-quantified based on the use of the internal standard method (Figure 1). What is remarkable, is that volatile compounds’ content (μg/L) of prickly pear juice was affected (*p* < 0.05) by its geographical origin (Table 2). Figure 1a–c represent typical gas chromatograms of prickly pear juice samples from the 3 investigated regions, pointing out the characteristic volatile compounds. Another important issue to note is that the specific alcohols and aldehydes identified in prickly pear juice have been previously reported to contribute to the distinctive aroma of numerous fruits and vegetables [26]. 

Before going any further, it is important to mention that the formation of flavors in fruits and vegetables may be the outcome of numerous mechanisms, including the lipoxygenase (LOX) pathway and autoxidation reactions [27,28,29]. In addition, a large number of volatile compounds may be formed in fruits and vegetables during maturation and handling procedures such as cutting, chewing, or during the application of mild heat treatment. In particular, fruits such as apples, pears, peaches, nectarines, apricots, and plums have been reported to have a typical green note when unripe [30,31]. 

In a previous study dealing with different prickly pear cultivars grown in the region of Paterno (Catania, Italy), Arena et al. [32] reported that the volatile profile recorded using HS-SPME/GC-MS was dominated by different amounts (μg/kg) of (E)-2-hexenal, (Z)-2-penten-1-ol, hexan-1-ol, (Z)-3-hexen-1-ol, (E)-2-hexen-1-ol, (E)-2-nonen-1-ol and (E,Z)-2,6-nonadien-1-ol, and trace amounts of (E)-2-nonenal and (E,Z)-2,6-nonadienal identified in a few samples. These findings are in agreement with the results of the present study.

Oumato et al. [33] using HS-SPME/GC-MS, reported that the dominant volatile compounds identified in three different prickly pear cultivars grown in the wider area of Morocco were 2-hexanal and 1-hexanol, among numerous others, also identified in the present study. 

The compound 2,6-Nonadienal has attracted great attention as the essence of cucumbers [34] but it was also found in freshly cut watermelon [35]. Compounds such as (E)-2-hexen-1-ol and (E,E)-2,4-decadienal have been reported to dominate the aroma of peaches (*P. persica*), nectarines (*P.persica* var. nucipersica), and sweet cherries (*P. avium*) [36,37]. On the other hand, 1-hexanol and 2-octenal contributed to the aroma of peas (*Pisum sativum*) [38]. A similar study on Egyptian prickly pear juice, or its blends with mandarin juice, highlighted that hexanal, nonanal, octanal, and (E)-2-hexenal (among other volatiles) contributed to its aroma [25]. This is in agreement with present results.

Another characteristic volatile compound, dl-Limonene, which represents the D-isomer of limonene, is a natural occurring volatile that is responsible for the characteristic flavor of citrus fruits. Limonene is formed from geranyl pyrophosphate, via cyclization of a neryl carbocation or its equivalent. The final step involves loss of a proton from the cation to form the alkene [39].

Table 2 also contains the orthonasal threshold value (OTV) for some volatile compounds available in the literature. OTV may be defined as the minimum amount of a compound that can be detected by the human nose. Aroma compounds are volatile compounds which are strongly perceived by the odor receptor sites of the olfactory tissue of the nasal cavity. These compounds directly reach the odor receptors when they “pass” through the nasal cavity (orthonasal detection) [42]. Based on available OTV literature data (Table 2), present results indicate that prickly pear juice is dominated by specific and undisputed aroma compounds. At this point, it should be mentioned that there is no study in the literature reporting data on the volatile profile of prickly pear juice prepared from prickly pear fruits grown in different areas of Peloponnese/Greece. Considering the above, the aroma of prickly pear juice is most probably the synergistic outcome of numerous volatiles. 

### 3.4. Valorization of Prickly Pear Juice Geographical Origin Based on Mineral Content Using LDA

MANOVA analysis identified the significant minerals that could be used for the geographical discrimination of prickly pear juice samples. The sixteen minerals were considered as the dependent variables, while the three regional zones (geographical origin) were taken as the independent variables. The two qualitative criteria of multivariate statistics namely Pillai’s trace = 1.694 (F = 6.571, df = 32, *p* < 0.001) and Wilks’ lambda = 0.022 (F = 6.469, df = 32, *p <* 0.001), showed that there was a statistically significant effect of prickly pear juice mineral content on the geographical origin of fruit juices. In particular, 7 of the 16 minerals (Table 3) were found to be significant (*p* < 0.05) for the geographical discrimination of prickly pear juices. These minerals were, then, subjected to LDA. In addition, the minimum tolerance level of the analysis was set at 0.001. Results showed that Fe did not pass the tolerance test. Therefore, it was excluded (SPSS program) a priori from the discriminant analysis. During LDA analysis two canonical discriminant functions were formed: Wilks’ lambda = 0.063, X^2^ = 80.320, df = 14, *p <* 0.001 for the first function and Wilks’ lambda = 0.348, X^2^ = 30.611, df = 6, *p <* 0.001, for the second. The first discriminant function recorded the higher eigenvalue (4.552) and canonical correlation of 0.905, accounting for 70.8% of total variance. The second discriminant function recorded a lower eigenvalue (1.874) and canonical correlation of 0.807, accounting for 29.2% of total variance. Both accounted for 100% of total variance.

**Figure 2:**
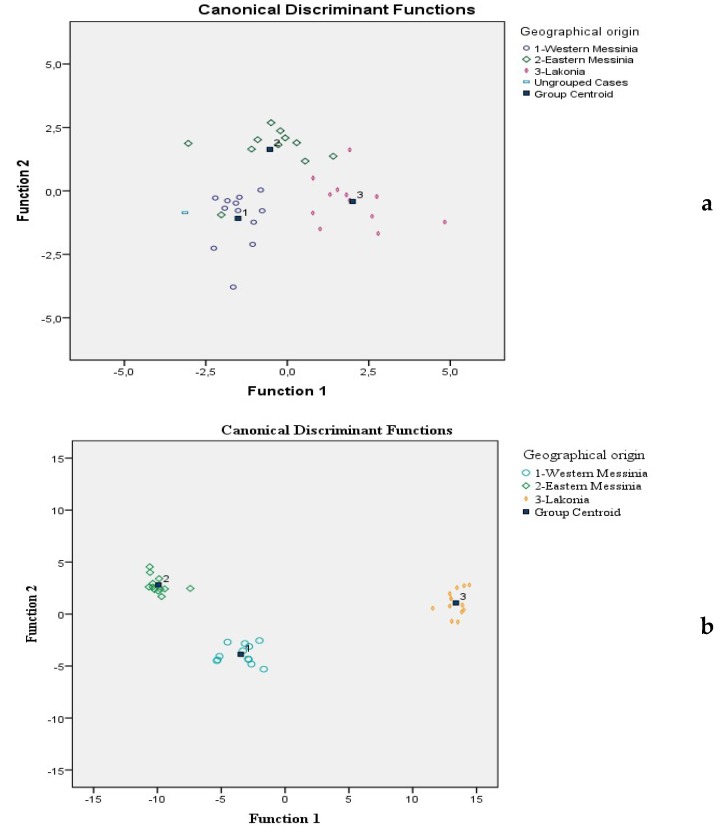
(**a**) Classification of prickly pear juice according to geographical origin based on 7 minerals and linear discriminant analysis (LDA). (**b**) Classification of prickly pear juice according to geographical origin based on 21 volatile compounds and LDA.

Prickly pear fruit juices are well separated, as shown in Figure 2a. The classification rate was 94.3% using the original, and 85.7% using the cross-validation method. The geographical classification rate was 91.7%, 81.8%, and 83.3% for Western Messinia, Eastern Messinia, and Lakonia, respectively (Table S10). The group centroid values, characteristic for each geographical origin, are also pointed out in Figure 2a. What should be defined, is that each centroid has two numbers which represent the coordinates. The abscissa is the first discriminant function and the ordinate is the second. The respective group centroid values were: (−1.899, 1.341), (−0.954, −1.834), and (2.774, 0.340) for the Western Messinia, Eastern Messinia, and Lakonia regions. The farther apart the means are, the less error there will be in classification [17]. 

Simpkins et al. [9] used inductively coupled plasma atomic emission spectroscopy (ICP-AES) and inductively coupled plasma mass spectrometry for the determination of minerals and trace elements (Al, B, Ba, Ca, Co, Cu, Fe, K, Li, Lu, Mg, Mn, Mo, Na, Ni, P, Rb, Si, Sr, Sn, Ti, V, and Zn) in Australian and Brazilian orange juice samples. Principal component analysis (PCA) in mineral and trace element contents resulted in a clear differentiation of orange juice samples. In another study, Pellerano et al. [13] used instrumental neutronic activation analysis (INAA) for the determination of Br, As, Na, Rb, La, Cr, Sc, Fe, Co, Zn, and Sb in lemon juice samples collected from three different geographical origins in the northwest region of Argentina. Application of PCA and LDA in the mineral content of lemon juice samples resulted in their correct classification according to geographical origin by 93.2%; higher by 7.5% compared to present results.

### 3.5. Valorization of Prickly Pear Juice Geographical Origin Based on Semi-Quantitative Data of Volatile Compounds Using LDA

Similarly, Pillai’s trace = 1.932 (F = 11.444, df = 50, *p <* 0.001) and Wilks’ lambda = 0.000 (F = 16.335, df = 50, *p <* 0.001) index values showed that there was a significant multivariable effect of volatile compounds on geographical origin of prickly pear juice. Twenty-two volatile compounds were found to be significant *(p <* 0.05) for the geographical discrimination of prickly pear juice samples. To avoid any misleading source, it should be stressed that 2-pentyl-furan, despite the significant differences (*p <* 0.05) among prickly pear juice samples according to geographical origin, was not included in the statistical analysis because it was categorized as a thermal artifact. Therefore, the semi-quantitative data of 21 volatile compounds was subjected to LDA.

Two canonical significant discriminant functions, as in the case of minerals, were formed: Wilks’ Lambda = 0.001, X^2^ = 159.643, df = 42, *p* < 0.001 for the first and Wilks’ Lambda = 0.103, X^2^ = 52.338, df = 20, *p <* 0.001 for the second function, respectively. The first discriminant function recorded the higher eigenvalue (105.215) and canonical correlation of 0.995, accounting for 92.3% of total variance. The second discriminant function recorded a much lower eigenvalue (8.733) and canonical correlation of 0.947, accounting for 7.7% of total variance. Both accounted for 100% of total variance which is a perfect rate. As it may be seen in Figure 2b, prickly pear fruit juices are clearly separated. The correct classification rate was 100% using the original and 88.9% using the cross-validation method. The geographical classification rate was 75%, 91.7%, and 100% for Western Messinia, Eastern Messinia and Lakonia, respectively (Table S11). Respective group centroid values were: (−3.469, −3.875), (−9.912, 2.803), and (13.381, 1.072) for Western Messinia, Eastern Messinia and Lakonia regions. The volatile compounds which served as markers of prickly pear juice geographical origin are listed in Table 4. However, differences in the volatile profile and as a consequence, in the classification rates obtained, may also be attributed to genetic factors (i.e., cultivar) [32,33], given the fact that wild-grown prickly pear cultivars from specific regions were used in the study.

In a relevant study, Reid et al. [10] used SPME-GC/MS for the determination of volatile compounds in apple juice prepared from different varieties. Further application of LDA in the volatile compounds data, resulted in the correct classification rate of apple juice samples by 87.5%. More recently, application of SPME-GC/MS and principal component analysis on the volatile compounds identified in tomato juice from Italian and Spanish markets resulted in the explanation of 68.61% of total variance among the samples analyzed [43].

### 3.6. Summary Regarding the Most Effective Predictors of Prickly Pear Juice Geographical Origin 

In Table 3 and Table 4 are listed with an asterisk (among other statistical analysis parameters) the standardized canonical discriminant function coefficients obtained in the selected models for every mineral and volatile compound according to prickly pear juice geographical origin. What is of great interest is that the higher the absolute value of a standardized canonical coefficient, the more significant the variable [17]. In both cases (analyses of minerals and volatile compounds) the first discriminant function was the one that differentiated best between prickly pear juice groups, given that it represented the highest variability (70.8% and 92.3%, respectively). However, the contribution of the second discriminant function is of considerable value, since both discriminant functions explained 100% of total variance. Based on the aforementioned, specific minerals and volatile compounds may effectively assist in the valorization of prickly pear juice from the Peloponnese Peninsula. 

## 4. Conclusions

Prickly pear juice prepared from wild cultivars grown in the region of Peloponnese is a good source of micro- and macro-minerals and may serve as a beneficial fruit beverage in the diet. On the other hand, prickly pear juice proved to have a balanced and distinctive aroma dominated by alcohols and aldehydes. A total of 7 minerals and 21 volatile compounds provided satisfactory classification rates of prickly pear juice samples according to geographical origin when subjected to LDA, and are proposed as “geographical origin indicators” of prickly pear juice from the Peloponnese Peninsula. The exploitation of prickly pear juice may support the local economy and contribute to the preparation of added value products of specific origin. 

## Figures and Tables

**Table 1:** Mineral content of prickly pear juice of different geographical origin.

Mineral (mg/kg)/Region	Al	B	Ca	Cu	Fe	K	Li	Mg	Mn	Na	Ni	P	Se	Si	Sn	Zn	TMC
Western Messinia (*n* = 12)																	
Average	0.26 ^a^	2.49 ^b^	83.78 ^c^	0.52 ^f^	1.21 ^g^	1869.70 ^h^	0.12 ^k^	93.92 ^l^	1.69 ^m^	21.36 ^o^	0.28 ^r^	127.52 ^t^	1.23 ^w^	0.24 ^y^	0.40 ^aa^	1.30 ^ac^	2206.07 ^ad^
±SD	0.26	0.50	9.68	0.35	0.79	205.64	0.10	11.80	2.04	17.41	0.25	17.70	2.05	0.46	0.47	0.56	214.77
Eastern Messinia (*n* = 12)																	
Average	0.45 ^a^	2.24 ^b^	59.61 ^d^	0.48 ^f^	1.36 ^g^	2175.97 ^i^	0.13 ^k^	100.20 ^l^	2.83 ^n^	48.14 ^p^	0.74 ^s^	186.13 ^u^	0.77 ^x^	0.08 ^z^	0.14 ^ab^	0.79 ^ad^	2580.11 ^ae^
±SD	1.15	0.45	20.72	0.21	2.33	161.25	0.07	10.18	0.96	25.94	0.21	27.57	0.42	0.18	0.29	0.22	206.06
Lakonia (*n* = 12)																	
Average	0.27 ^a^	2.63 ^b^	89.66 ^e^	0.67 ^f^	0.76 ^g^	2398.60 ^j^	0.10 ^k^	108.50 ^l^	1.39 ^m^	33.87 ^q^	0.35 ^r^	188.53 ^v^	0.70 ^x^	0.24 ^y^	0.37 ^aa^	1.27 ^ac^	2827.98 ^af^
± SD	0.38	0.81	33.85	0.39	0.70	252.36	0.10	14.74	2.43	31.10	0.48	28.94	0.38	0.34	0.40	0.33	310.65
LOD	4.90	0.32	4.06	1.40	1.96	1.47	0.12	5.18	0.80	1.45	0.74	1.50	1.49	0.11	8.84	0.40	
LOQ	14.70	1.06	12.17	4.19	5.87	5.41	0.40	15.53	2.40	5.47	2.21	5.00	4.48	0.36	26.53	1.20	
CV (*n* = 36)	2.15	0.25	0.34	0.59	1.31	0.14	0.78	0.13	0.99	0.79	0.84	0.23	1.35	1.83	1.32	0.40	

N: number of prickly pear juice samples. SD: standard deviation values of three replicates (*n* = 3). Different letters in each column indicate statistically significant differences (*p* < 0.05). LOD: limit of detection (μg/kg). LOQ: limit of quantification (μg/kg). CV: coefficient of variation; defined as the ratio of standard deviation to the average, often expressed as a percentage. TMC: total mineral content (mg/kg).

**Table 2:** Volatile compounds of prickly pear juice tentatively identified and semi-quantified according to geographical origin.

RT (min)	Volatile Compounds (μg/L)	KI ^a^	Western Messinia (Avg ± SD)	Eastern Messinia (Avg ± SD)	Lakonia (Avg ± SD)	Method of Identification	MS Qualification (%)	Orthonasal Threshold (μg/L) ^b^	Odour Note ^c^
	*Alcohols*								
11.66	1-Pentanol	702	192.54 ± 90.69	178.18 ± 54.94	314.94 ± 105.62	MS	90	0.0055-305	Fermented, green
15.27	3-Hexen-1-ol	787	32.26 ± 15.86	41.02 ± 31.12	47.81 ± 21.01	MS	94	na	Woody, green, leafy
15.89	2-Hexen-1-ol	802	2486.91 ± 593.02	2681.84 ± 1280.63	3022.18 ± 1101.85	MS/KI	91	na	Green, fruity, leafy
16.02	1-Hexanol	804	2372.75 ± 1341.20	3885.41 ± 941.84	3429.98 ± 1149.45	MS/KI	90	500-2500	Green, fruity
16.86	3,5-Hexadien-1-ol	823	120.61 ± 57.82	189.66 ± 53.18	180.42 ± 67.70	MS/KI	92	na	
24.79	1-Octanol	999	ni	ni	55.27 ± 46.57	MS/KI	90	190	Herbal, green, penetrating
28.67	2,6 Nonadien-1-ol	1090	318.22 ± 265.20	270.39 ± 222.85	797.17 ± 337.37	MS/KI	91	na	Green, cucumber-like
28.75	2-Nonen-1-ol	1091	443.17 ± 240.80	564.97 ± 124.63	921.67 ± 240.39	MS/KI	92	na	Fatty
	*Aldehydes*								
7.63	2-Butenal	520	ni	ni	41.58 ± 19.44	MS	90	na	Floral
9.13	Pentanal	620	ni	ni	19.87 ± 18.71	MS	86	na	Bready, fruity, berry-like
13.17	Hexanal	737	431.69 ± 218.41	288.86 ± 241.71	692.64 ± 156.50	MS	96	9.18-10.50	Green, grassy, floral
15.51	2-Hexenal	793	512.87 ± 263.34	627.65 ± 411.57	1129.38 ± 258.88	MS	96	24.2	Soapy, fatty, green
17.63	Heptanal	840	26.20 ± 17.26	ni	43.79 ± 46.45	MS/KI	90	na	Fruity, oily-greasy
18.14	2,4-Hexadienal	851	ni	ni	27.72 ± 23.07	MS/KI	92	na	Green, fruity, waxy
20.11	2-Heptenal	894	29.28 ± 28.21	ni	55.93 ± 22.71	MS/KI	97	na	Green, fatty, oily, fruity
24.48	2-Octenal	992	64.02 ± 49.55	ni	181.08 ± 48.33	MS/KI	91	na	Sweet, green, fatty, brothy
26.34	Nonanal	1035	86.00 ± 35.76	96.35 ± 20.97	187.25 ± 58.03	MS/KI	91	2.53-5.00	Soapy, floral
28.37	2,6-Nonadienal	1082	78.21 ± 70.68	80.75 ± 68.90	378.80 ± 152.75	MS/KI	90	na	Cucumber-like, green
34.37	2,4-Decadienal	1243	ni	ni	54.09 ± 47.75	MS/KI	93	0.2	Fatty, waxy, green
	*Hydrocarbons*								
6.95	2,4-Hexadiene	473	ni	ni	12.66 ± 13.81	MS	90	na	na
21.52	1-Decene	926	ni	75.67 ± 65.39	ni	MS/KI	95	6.45	Pleasant
21.89	Decane	934	ni	22.57 ± 20.78	ni	MS/KI	93	na	na
29.73	1-Dodecene	1116	ni	64.01 ± 60.12	ni	MS/KI	95	na	na
	*Terpenoids*								
23.56	dl-Limonene	971	23.07 ± 49.07	14.85 ± 9.83	ni	MS/KI	98	0.0018-0.31	Lemon, citrus
	*Furan derivatives*								
21.58	Furan, 2-pentyl-	927	40.71 ± 35.39	ni	109.03 ± 81.45	MS/KI	91	10.06	Strong

RT: retention time (min). ^a^ KI: Kovats index. Avg ±SD: average value ±standard deviation. MS: mass spectra. ni: not identified. na: not available. ^b^: Berger [26], AIHA [40]. ^c^: Berger [26], Burdock [41].

**Table 3:** Contribution of minerals to the canonical discriminant function structure matrix.

Minerals	Wilks’ Lambda	F	df1	df2	*p*	Function 1	Function 2
Ca	0.748	5.397	2	32	0.0010	0.132	0.371 *
K	0.458	18.938	2	32	<0.001	0.463 *	−0.334
Mg	0.797	4.070	2	32	0.027	0.230 *	−0.086
Na	0.770	4.768	2	32	0.015	0.025	−0.397 *
Ni	0.746	5.439	2	32	0.009	−0.047	−0.419 *
P	0.428	21.397	2	32	<0.001	0.381	−0.600 *
Zn	0.704	6.741	2	32	0.004	0.081	0.457 *

F: function value, df: degrees of freedom, *p:* level of significance. Pooled within-groups correlations between discriminating variables and standardized canonical discriminant functions. Variables ordered by absolute size of correlation within function. * Largest absolute correlation between each variable and any discriminant function.

**Table 4:** Contribution of volatile compounds to the canonical discriminant function structure matrix.

Volatile Compounds (μg/L)	Wilks’ Lambda	F	df1	df2	*p*	Function 1	Function 2
2-Butenal	0.231	54.909	2	33	<0.001	0.171 **	0.165
2,4-Decadienal	0.517	15.401	2	33	<0.001	0.091 **	0.088
Decanal	0.538	14.158	2	33	<0.001	−0.064	0.220 **
Decene	0.507	16.070	2	33	<0.001	−0.069	0.234 *
1-Dodecene	0.548	13.604	2	33	<0.001	−0.063	0.215 *
Heptanal	0.699	7.120	2	33	0.003	0.060	−0.079 **
2-Heptenal	0.434	21.488	2	33	<0.001	0.107 **	−0.107
2,4-Hexadienal	0.488	17.326	2	33	<0.001	0.096 **	0.093
2,4-Hexadiene	0.620	10.094	2	33	<0.001	0.073 **	0.071
3,5-Hexadien-1-ol	0.778	4.703	2	33	0.016	0.007	0.179 **
Hexanal	0.588	11.554	2	33	<0.001	0.081 **	−0.024
1-Hexanol	0.753	5.410	2	33	0.009	−0.003	0.194 **
2-Hexenal	0.566	12.657	2	33	<0.001	0.077	0.128 **
2,6-Nonadienal	0.337	32.509	2	33	<0.001	0.132 **	0.131
2,6-Nonadien-1-ol	0.558	13.067	2	33	<0.001	0.085 **	0.057
Nonanal	0.429	21.969	2	33	<0.001	0.105	0.139 **
2-Nonen-1-ol	0.493	16.955	2	33	<0.001	0.086	0.170 **
1-Octanol	0.494	16.903	2	33	<0.001	0.095 **	0.092
2-Octenal	0.207	63.348	2	33	<0.001	0.190 **	−0.056
Pentanal	0.549	13.541	2	33	<0.001	0.085 **	0.082
Pentanol	0.645	9.079	2	33	0.001	0.071 **	0.044

F: function value, df: degrees of freedom, *p:* level of significance. Pooled within-groups correlations between discriminating variables and standardized canonical discriminant functions. Variables ordered by absolute size of correlation within function. ** Largest absolute correlation between each variable and any discriminant function.

## Supplementary Materials

The following are available online at https://www.mdpi.com/2304-8158/8/4/123/s1, Table S1: Linearity of the calibration curves used for the quantification of minerals in soil samples, Table S2: Linearity of the calibration curves used for the quantification of minerals in prickly pear juice samples, Table S3a: Mineral recoveries in soil samples at 2 mg/kg, Table S3b: Mineral recoveries in soil samples at 200 mg/kg, Table S4: Mineral recoveries in prickly pear juice samples at different spiking concentrations, Table S5: Limit of detection (LOD) and limit of quantification (LOQ)* in soil samples, Table S6: Limit of detection (LOD) and limit of quantification (LOQ) (μg/kg) in fruit juice samples, Table S7: Mineral content (mg/kg) of prickly pear juice samples according to geographical origin, Table S8: SPME-GC/MS method optimization/development, Table S9: Mineral content (mg/L) of soil samples according to prickly pear geographical origin, Table S10: Discriminatory ability of the developed LDA model for the classification of prickly pear juice according to geographical origin based on 7 minerals, Table S11: Discriminatory ability of the developed LDA model for the classification of prickly pear juice according to geographical origin based on 21 volatile compounds, Figure S1: A typical gas chromatogram of a prickly pear juice mixture of the 3 regions during method optimization. TEST1, TEST2, and TEST 4 refer to methods 1, 2, 3 (Table S8), Figure S2: A typical gas chromatogram of a prickly pear juice mixture of the 3 regions during method optimization. TEST5, TEST6, and TEST7 refer to method 3 (Table S8), Figure S3: A typical gas chromatogram of a prickly pear juice mixture of the 3 regions during method optimization. TEST8, TEST9, and TEST10 refer to method 3 (Table S8), Figure S4: A typical gas chromatogram of a prickly pear juice mixture of the 3 regions during method optimization. TEST11, TEST12, and TEST13 refer to method 3 (Table S8), Figure S5: A typical gas chromatogram of a prickly pear juice mixture of the 3 regions during method optimization. TEST14, TEST15, and TEST17 refer to method 3 (Table S8), Figure S6: A typical gas chromatogram of a prickly pear juice mixture of the 3 regions during method optimization. TEST7, TEST10, and TEST13 refer to method 3 (Table S8), Figure S7: A typical gas chromatogram of a prickly pear juice mixture of the 3 regions during method optimization. TEST13 and TEST17 refer to method 3 (Table S8), Figure S8: A typical gas chromatogram of a prickly pear juice mixture of the 3 regions during method optimization. TEST20 and TEST22 refer to method 3 (Table S8).
