# Supplementary material for: Valorization of Prickly Pear Juice Geographical Origin Based on Mineral and Volatile Compound Contents Using LDA
Source: Foods. 2019 Apr 15;8(4):123. doi: 10.3390/foods8040123 (PMC6518022; doi:10.3390/foods8040123)
Supplement: Supplementary file 1 [file foods-08-00123-s001.pdf]

Supplementary Table 1 (ST1). Linearity of the calibration curves used for the quantification of minerals in soil samples.

Minerals	Intercept, A_0	Slope, A_1	Correlation Coefficient (R^2)
Al	0.117	0.093	0.9987
B	0.042	2.707	0.9996
Ba	−5.238	94.192	0.9999
Be	0.024	4435.800	0.9999
Co	0,199	251.199	0.9996
Cr	0.003	0.600	0.9999
Cu	−0.023	3.947	1.0000
Fe	0.028	1.561	0,9999
K	0.354	2.547	0.9987
Li	0.104	0.678	0.9999
Mg	0.006	2.507	1.0000
Mn	−0.098	7.389	0,9999
Mo	−3.072	98.992	0.9988
Na	6.478	8745	0.9997
Ni	0.685	102.439	0.9991
P	1.300	3.500	0.9990
Sb	−0.135	6.199	0.9883
Se	0.120	8.250	0.9979
Si	−0.009	0.296	0.9995
Sn	0,108	16.405	0.9996
Ti	0.267	59,469.1	0.9988
Tl	0.008	27,8433.6	0.9997
V	−0.002	0.276	0.9998
Zn	76.790	451.360	0.9967

Supplementary Table 2 (ST2). Linearity of the calibration curves used for the quantification of minerals in prickly pear juice samples.

Minerals	Intercept, A_0	Slope, A_1	Correlation coefficient (R^2)
Al	0.055	0.899	1.000
B	0.042	2.707	0.999
Ba	–5.238	94.192	0.999
Be	3.639	–10.830	0.997
Ca	0.548	58.470	0.996
Co	0.181	0.008	1.000
Cr	0.021	–0.623	1.000
Cu	0.097	1.955	1.000
Fe	0.032	1.401	0.999
K	2.547	1.789	0.999
Li	0.874	2.698	1.000
Mg	0.006	2.507	1.000
Mn	0.166	0.542	1.000
Mo	0.054	-0.828	1.000
Na	2.478	5.780	0.995
Ni	0.087	–0.006	1.000
P	1.300	3.500	0.999
Sb	0.016	0.501	0.999
Se	0.005	–0.140	0.999
Si	–0.009	0.296	0.999
Sn	0.004	1.385	0.999
Ti	0.066	0.548	1.000
Tl	0.012	0.165	1.000
V	0.041	0.758	1.000
Zn	0.343	0.781	1.000

Recoveries in soil samples at different spiking concentrations.

Supplementary Table 3 (ST3a). Mineral recoveries in 2 mg/kg.

Recovery (%)	Al	B	Ba	Be	Ca	Co	Cr	Cu	Fe	K	Li	Mg	Mn	Mo	Na	Ni	P	Sb	Se	Si	Sn	Ti	TI	V	Zn
Trial 1	87.0	86.0	88.4	95.8	88.4	80.0	72.3	83.7	96.2	87.4	88.4	88.4	75.7	77.2	88.7	84.7	98.2	88.0	84.3	78.9	87.5	87.4	79.9	72.2	97.4
Trial 2	88.7	88.9	83.5	99.1	88.7	80.6	75.5	88.2	89.7	88.7	89.7	89.2	80.9	82.7	88.7	82.8	102.6	88.7	82.9	75.9	87.6	89.4	81.4	75.5	97.2
Trial 3	86.7	90.0	91.5	99.2	91.4	80.6	76.8	89.7	76.2	90.7	99.1	88.7	82.5	83.8	84.5	82.4	107.2	88.1	67.4	71.6	88.4	99.1	81.5	76.6	95.7
Trial 4	90.1	93.6	99.7	100.2	90.8	79.3	79.5	102.3	85.4	98.5	99.0	88.7	90.7	85.6	91.2	81.9	100.2	84.1	68.9	84.5	91.2	91.4	81.8	80.6	90.4
Trial 5	99.4	94.6	85.4	100.0	92.5	78.9	79.4	102.8	83.4	99.7	97.4	87.4	91.2	85.8	99.5	83.1	100.5	83.8	68.4	83.7	99.5	92.4	81.3	80.7	89.8
Trial 6	93.4	94.9	87.4	100.7	94.8	78.8	80.0	103.1	70.4	94.3	97.6	84.7	92.0	86.1	94.3	83.1	103.3	83.5	68.4	80.8	94.3	97.9	81.5	81.4	90.1
**Average**	**90.9**	**93.1**	**89.3**	**104.7**	**91.1**	**83.0**	**82.8**	**99.3**	**83.5**	**93.2**	**95.1**	**87.9**	**91.4**	**89.7**	**91.1**	**83.0**	**102.0**	**88.1**	**73.7**	**79.2**	**91.4**	**92.9**	**85.5**	**83.1**	**93.4**

Recovery (%); average value of six replicates (n = 6).

Supplementary Table 3 (ST3b) Mineral recoveries in 200 mg/kg.

Recovery (%)	Al	B	Ba	Be	Ca	Co	Cr	Cu	Fe	K	Li	Mg	Mn	Mo	Na	Ni	P	Sb	Se	Sn	Si	Ti	TI	V	Zn
Trial 1	96.8	82.5	105.3	83.7	84.8	92.4	79.5	88.9	89.7	96.7	95.0	106.9	79.4	84.8	94.0	87.3	73.9	87.0	81.1	79.9	80.2	84.0	83.5	78.1	98.0
Trial 2	98.7	84.6	104.9	84.9	85.5	92.7	82.2	91.4	91.9	96.4	93.4	106.9	82.5	83.9	100.4	87.9	82.6	86.3	81.5	80.0	81.4	84.4	84.2	80.3	97.4
Trial 3	99.7	85.0	105.5	85.2	86.1	93.2	82.7	91.4	92.7	98.4	95.1	104.7	83.0	83.9	97.4	89.3	81.4	87.7	81.4	80.4	82.1	84.8	84.1	80.7	97.7
Trial 4	99.4	85.5	104.1	83.7	85.2	93.3	83.1	91.3	92.6	97.9	95.1	106.0	83.4	83.7	99.1	88.8	80.1	88.3	80.0	91.3	111.4	89.4	82.1	80.9	95.9
Trial 5	99.5	85.7	104.3	84.4	85.3	89.3	83.5	92.0	92.3	95.3	92.7	101.6	83.9	83.6	86.4	88.9	78.9	88.3	80.0	90.9	112.2	89.7	82.3	81.0	96,1
Trial 6	99.9	85.6	104.0	84.4	85.5	90.0	83.6	91.7	92.6	95.8	92.5	103.7	84.1	83.6	87.4	89.0	75.1	87.8	79.8	91.0	111.6	89.3	82.2	81.1	96.0
**Average**	**99.0**	**85.0**	**104.7**	**84.5**	**85.4**	**91.8**	**82.7**	**91.3**	**91.9**	96.8	**94.0**	**105.0**	**83.0**	**83.9**	**93.6**	**88.5**	**75.7**	**87.6**	**80.4**	**85.6**	**102.4**	**87.8**	**83.0**	**80.4**	**96.9**

Recovery (%); average value of six replicates (n = 6).

Supplementary Table 4 (ST4). Mineral recoveries in prickly pear juice samples at different spiking concentrations.

Mineral	**Al**	**B**	**Ba**	**Be**	**Ca**	**Co**	**Cr**	**Cu**	**Fe**	**K**	**Li**	**Mg**	**Mn**	**Mo**	**Na**	**Ni**	**P**	**Sb**	**Se**	**Si**	**Sn**	**Ti**	**Tl**	**V**	**Zn**
R (%)	**0.5 ppm**	**0.5 ppm**	**0.5 ppm**	**0.5 ppm**	**0.5 ppm**	**0.5 ppm**	**0.5 ppm**	**0.5 ppm**	**0.5 ppm**	**0.5 ppm**	**0.5 ppm**	**0.5 ppm**	**0.5 ppm**	**0.5 ppm**	**0.5 ppm**	**0.5 ppm**	**0.5 ppm**	**0.5 ppm**	**0.5 ppm**	**0.5 ppm**	**0.5 ppm**	**0.5 ppm**	**0.5 ppm**	**0.5 ppm**	**0.5 ppm**
Trial 1	112.1	98.4	108.7	102.0	98.4	100.6	94.1	81.3	85.2	101.2	102.4	100.4	88.7	103.9	89.7	97.4	96.4	97.2	114.8	113.0	98.7	101.4	100.4	91.0	100.4
Trial 2	115.8	99.2	108.6	102.5	97.4	100.8	90.3	82.4	92.6	103.5	108.7	104	94.3	104.3	88.4	97.7	94.8	96.6	115.9	113.6	99.2	100.4	104.7	92.4	100.7
Trial 3	112.8	97.4	106.6	101.8	99.8	100.1	92.4	82.2	82.7	99.4	99.4	101.5	93.5	102.9	87.6	97.1	93.7	99.9	116.3	113.7	97.6	100.8	103.5	91.0	98.7
Trial 4	112.5	97	107.3	102.2	100.0	99.7	92.8	80.6	83	89	98.7	100.4	96.4	102.8	88.4	97.4	99.2	96.3	115.4	113.2	96.4	102.4	102.7	89.5	97.4
Trial 5	111.8	98.1	108.2	101.8	98.3	100.5	94.9	81.3	84.7	94.7	97.4	87.4	97.4	102.8	82.1	97.5	89.4	98.9	115.9	113.1	91.2	103	101.4	89.1	96.6
**Avg**	**113.0**	**98.02**	**107.9**	**102.0**	**98.8**	**100.3**	**92.9**	**81.6**	**85.6**	**97.56**	**101.32**	**98.74**	**94.06**	**103.4**	**87.24**	**97.4**	**94.7**	**97.8**	**115.7**	**113.4**	**96.62**	**101.6**	**102.5**	**90.6**	**98.76**
R (%)	**50 ppb**	**50 ppb**	**50 ppb**	**50 ppb**	**50 ppb**	**50 ppb**	**50 ppb**	**50 ppb**	**50 ppb**	**50 ppb**	**50 ppb**	**50 ppb**	**50 ppb**	**50 ppb**	**50 ppb**	**50 ppb**	**50 ppb**	**50 ppb**	**50 ppb**	**50 ppb**	**50 ppb**	**50 ppb**	**50 ppb**	**50 ppb**	**50 ppb**
Trial 1	116.9	87.1	80.4	104.2	99.7	89.6	97.4	98.0	102.5	71.5	91.7	95.1	99.4	95.6	100.4	88.9	90.4	91.1	87.7	78.2	117.2	87.9	88.4	91.1	110.0
Trial 2	85.2	83.2	81.5	102.9	98.4	90.6	97.9	95.7	99.4	72.3	92.7	96.2	99.8	96.2	100.8	90.2	91.7	92.1	88.3	78.6	112.6	88.5	86.4	91.8	110.8
Trial 3	85.2	84.2	97.0	103.4	96.1	89.6	95.3	96.1	98.9	75.2	91.0	93.4	97.8	95.3	100.0	88.8	99.7	87.0	88.1	75.2	114.6	84.4	89.4	90.0	103.6
Trial 4	108.9	94.0	86.5	102.8	91.4	92.1	96.4	98.6	98.4	75.3	91.4	107.6	99.7	95.4	101.6	91.2	104.5	91.9	85.5	79.0	116.5	86.8	89.4	89.6	104.8
Trial 5	109.4	95.3	70.3	102.8	93.5	92.3	98.2	98.3	97.5	73.3	94.2	97.0	99.1	97.5	103.2	91.3	101.4	89.3	89.6	84.2	114.3	86.9	91.5	92.0	105.1
**Avg**	103.7	89.5	71.7	103.3	95.8	90.6	97.1	97.4	99.9	73.5	92.5	97.7	98.9	96.3	101.9	89.9	97.5	90.2	87.4	79.3	115.1	86.4	89.0	91.1	106.3

R: Recovery (%); avg: average value of five replicates (n = 5); ppm: parts per million (mg/kg); ppb: parts per billion (μg/kg).

Supplementary Table 5 (ST5). Limit of detection (LOD) and limit of quantification (LOQ)* in soil samples.

	**Al**	**B**	**Ba**	**Be**	**Ca**	**Co**	**Cr**	**Cu**	**Fe**	**K**	**Li**	**Mg**	**Mn**	**Mo**
**LOD (mg/L)**	0.027	0.013	0.001	0.001	0.001	0.028	0.019	0.003	0.025	0.004	0.001	0.001	0.003	0.020
**LOQ (mg/L)**	0.080	0.080	0.080	0.080	0.080	0.080	0.080	0.080	0.080	0.080	0.080	0.080	0.080	0.080

	**Na**	**Ni**	**P**	**Sb**	**Se**	**Si**	**Sn**	**Ti**	**Tl**	**V**	**Zn**
**LOD (mg/L)**	0.002	0.002	0.027	0.004	0.020	0.028	0.001	0.008	0.015	0.002	0.023
**LOQ (mg/L)**	0.080	0.080	0.080	0.080	0.080	0.080	0.080	0.080	0.080	0.080	0.080

*LOD and LOQ are multiplied with the dilutions made, so the resulting values are expressed as mg/L.

Supplementary Table 6 (ST6). Limit of detection (LOD) and limit of quantification (LOQ) (μg/kg) in fruit juice samples.

Prickly Pear Juice Samples	Al	B	Ba	Be	Ca	Co	Cr	Cu	Fe	K	Li	Mg	Mn	Mo	Na	Ni	P	Sb	Se	Si	Sn	Ti	Tl	V	Zn
LOD (μg/kg)	4.90	0.32	1.04	0.69	4.06	0.42	1.59	1.40	1.96	1.47	0.12	5.18	0.80	1.01	1.45	0.74	1.50	0.52	1.49	0.11	8.84	1.06	2.40	0.39	0.40
LOQ (μg/kg)	14.70	1.06	3.43	2.06	12.17	1.27	4.78	4.19	5.87	5.41	0.40	15.53	2.40	3.03	5.47	2.21	5.00	1.57	4.48	0.36	26.53	3.19	7.21	1.18	1.20

Supplementary Table 7 (ST 7). Mineral content (mg/kg) of prickly pear juice samples according to geographical origin.

Region	Al	B	Ba	Be	Ca	Co	Cr	Cu	Fe	K	Li	Mg	Mn
Western Messinia 1	0.10	2.62	0.04	0.00	83.98	0.00	0.00	0.27	1.02	1725.63	0.24	103.16	1.74
Western Messinia 2	0.27	3.77	0.00	0.00	88.91	0.00	0.00	1.07	0.50	1603.64	0.00	118.66	1.26
Western Messinia 3	0.12	1.91	0.05	0.00	68.79	0.00	0.00	0.29	1.25	1650.19	0.13	88.48	1.47
Western Messinia 4	0.27	2.50	0.00	0.00	88.84	0.00	0.00	0.29	0.42	2289.74	0.20	102.07	0.40
Western Messinia 5	0.14	2.23	0.00	0.00	82.04	0.00	0.00	0.29	1.31	2099.17	0.17	84.36	0.89
Western Messinia 6	0.09	2.24	0.02	0.00	64.31	0.00	0.00	0.31	3.41	1646.57	0.19	102.47	1.54
Western Messinia 7	0.33	2.22	0.04	0.00	81.91	0.00	0.00	0.30	1.51	1780.46	0.12	98.11	1.66
Western Messinia 8	0.09	2.12	0.00	0.00	86.75	0.00	0.00	0.24	1.40	1934.03	0.18	75.62	8.04
Western Messinia 9	0.00	2.98	0.05	0.00	97.06	0.00	0.00	0.96	0.74	2019.73	0.00	94.99	0.83
Western Messinia 10	0.20	2.64	0.08	0.00	84.76	0.00	0.00	0.88	0.73	1869.89	0.00	90.68	0.79
Western Messinia 11	0.93	2.55	0.09	0.00	96.99	0.00	0.00	1.04	0.79	1841.88	0.00	87.49	0.85
Western Messinia 12	0.57	2.13	0.04	0.00	81.01	0.00	0.00	0.25	1.43	1975.51	0.27	80.97	0.84
AVG	***0.26***	***2.49***	***0.03***	***0.00***	***83.78***	***0.00***	***0.00***	***0.52***	***1.21***	***1869.70***	***0.12***	***93.92***	***1.69***
±SD	***0.26***	***0.50***	***0.03***	***0.00***	***9.68***	***0.00***	***0.00***	***0.35***	***0.79***	***205.64***	***0.10***	***11.80***	***2.04***
Eastern Messinia 1	0.00	2.14	0.06	0.00	52.85	0.00	0.00	0.47	0.38	2080.21	0.08	94.75	2.29
Eastern Messinia 2	0.21	1.75	0.04	0.00	57.48	0.00	0.00	0.52	0.12	2179.06	0.08	102.80	2.78
Eastern Messinia 3	0.00	2.22	0.07	0.00	66.17	0.00	0.00	0.47	0.49	2169.21	0.17	101.33	2.65
Eastern Messinia 4	0.00	2.63	0.05	0.00	70.18	0.00	0.00	0.39	4.96	2054.96	0.20	101.40	3.71
Eastern Messinia 5	0.22	2.04	0.00	0.00	9.40	0.00	0.00	0.35	0.00	2105.42	0.06	89.42	1.99
Eastern Messinia 6	4.05	2.48	0.06	0.00	92.57	0.00	0.00	0.48	0.73	2537.31	0.08	102.49	4.74
Eastern Messinia 7	0.00	3.34	0.04	0.00	57.89	0.00	0.00	1.10	7.43	2142.71	0.00	119.95	3.20
Eastern Messinia 8	0.07	1.94	0.04	0.00	80.61	0.00	0.00	0.20	0.62	1913.14	0.21	78.06	0.78
Eastern Messinia 9	0.70	1.95	0.05	0.00	64.76	0.00	0.00	0.44	0.66	2359.42	0.11	109.15	2.68
Eastern Messinia 10	0.00	1.89	0.05	0.00	65.66	0.00	0.00	0.43	0.45	2073.26	0.23	100.69	2.97
Eastern Messinia 11	0.00	2.51	0.05	0.00	58.17	0.00	0.00	0.46	0.51	2277.74	0.18	98.22	2.85
Eastern Messinia 12	0.09	1.93	0.04	0.00	39.57	0.00	0.00	0.44	0.00	2219.23	0.18	104.09	3.36
AVG	***0.45***	***2.24***	***0.05***	***0.00***	***59.61***	***0.00***	***0.00***	***0.48***	***1.36***	***2175.97***	***0.13***	***100.20***	***2.83***
±SD	***1.15***	***0.45***	***0.02***	***0.00***	***20.72***	***0.00***	***0.00***	***0.21***	***2.33***	***161.25***	***0.07***	***10.18***	***0.96***
Lakonia 1	0.39	1.80	0.00	0.00	72.67	0.00	0.00	0.49	0.49	2481.89	0.09	102.66	0.28
Lakonia 2	1.20	4.29	0.17	0.00	128.13	0.00	0.00	1.27	1.25	2272.35	0.00	128.45	0.50
Lakonia 3	0.00	3.38	0.00	0.00	82.88	0.00	0.00	1.16	0.16	2039.60	0.00	98.64	1.17
Lakonia 4	0.52	2.37	0.10	0.00	69.18	0.00	0.00	0.38	0.48	2516.56	0.29	115.27	2.34

Lakonia 5	0.00	2.46	0.00	0.00	79.69	0.00	0.00	0.25	0.29	2288.35	0.25	103.98	0.90
Lakonia 6	0.00	3.32	0.00	0.00	78.09	0.00	0.00	1.15	0.24	2266.52	0.00	123.94	0.42
Lakonia 7	0.34	2.09	0.00	0.00	53.97	0.00	0.00	0.40	2.68	2290.55	0.18	92.41	0.37
Lakonia 8	0.67	2.08	0.00	0.00	72.43	0.00	0.00	0.43	0.56	2520.14	0.06	100.46	0.39
Lakonia 9	0.09	2.24	0.00	0.00	86.26	0.00	0.03	0.36	1.26	2405.22	0.10	98.89	0.52
Lakonia 10	0.00	2.02	0.00	0.00	82.65	0.00	0.00	0.46	0.67	2245.66	0.16	99.77	0.60
Lakonia 11	0.00	3.67	0.16	0.00	181.87	0.00	0.03	1.18	0.61	3073.27	0.00	139.46	8.87
Lakonia 12	0.05	1.90	0.00	0.00	88.06	0.00	0.00	0.53	0.42	2383.15	0.03	98.23	0.32
AVG	***0.27***	***2.63***	***0.04***	***0.00***	***89.66***	***0.00***	***0.00***	***0.67***	***0.76***	***2398.60***	***0.10***	***108.51***	***1.39***
±SD	***0.38***	***0.81***	***0.07***	***0.00***	***33.85***	***0.00***	***0.01***	***0.39***	***0.70***	***252.36***	***0.10***	***14.74***	***2.43***
CV (N = 36)	*2.15*	*0.25*	*1.11*	*0.00*	*0.34*	*0.00*	*4.22*	*0.59*	*1.31*	*0.14*	*0.78*	*0.13*	*0.99*

Supplementary Table 7. (Continued).

Region	Mo	Na	Ni	P	Sb	Se	Si	Sn	Ti	Tl	V	Zn	TMC
Western Messinia 1	0.00	33.75	0.18	119.08	0.00	1.00	0.00	0.24	0.00	0.00	0.00	0.83	2073.87
Western Messinia 2	0.00	0.00	0.16	119.28	0.00	0.33	0.51	0.67	0.00	0.04	0.00	0.98	1940.06
Western Messinia 3	0.00	37.90	0.15	129.23	0.00	0.84	0.00	0.00	0.00	0.00	0.00	0.82	1981.61
Western Messinia 4	0.00	11.02	0.96	179.37	0.00	0.79	0.00	0.35	0.00	0.00	0.00	1.66	2678.88
Western Messinia 5	0.00	31.99	0.10	132.47	0.00	1.03	0.00	0.00	0.00	0.00	0.00	1.00	2437.16
Western Messinia 6	0.00	42.13	0.18	124.02	0.00	0.00	0.00	0.06	0.00	0.00	0.00	0.89	1988.45
Western Messinia 7	0.00	33.03	0.18	126.84	0.00	7.67	0.00	0.00	0.00	0.00	0.00	0.94	2135.31
Western Messinia 8	0.00	35.45	0.11	122.15	0.00	0.85	0.00	0.20	0.00	0.00	0.00	0.98	2268.20
Western Messinia 9	0.00	0.00	0.39	130.36	0.00	0.44	0.23	1.25	0.00	0.00	0.00	1.84	2351.84
Western Messinia 10	0.00	0.00	0.38	111.03	0.00	0.46	0.27	1.03	0.00	0.00	0.00	1.84	2165.67
Western Messinia 11	0.00	0.00	0.48	111.25	0.00	0.43	0.31	1.05	0.00	0.00	0.00	2.59	2148.71
Western Messinia 12	0.00	31.11	0.11	125.11	0.00	0.95	1.59	0.00	0.00	0.00	0.00	1.18	2303.07
AVG	***0.00***	***21.36***	***0.28***	***127.52***	***0.00***	***1.23***	***0.24***	***0.40***	***0.00***	***0.00***	***0.00***	***1.30***	**2206.07**
±SD	***0.00***	***17.41***	***0.25***	***17.70***	***0.00***	***2.05***	***0.46***	***0.47***	***0.00***	***0.01***	***0.00***	***0.56***	**214.77**
Eastern Messinia 1	0.00	56.75	0.63	146.27	0.00	1.05	0.00	0.00	0.00	0.00	0.00	0.45	2438.38
Eastern Messinia 2	0.00	74.87	0.57	191.18	0.00	1.16	0.00	0.06	0.00	0.00	0.00	0.83	2613.51
Eastern Messinia 3	0.00	50.22	0.92	206.46	0.00	0.00	0.00	0.00	0.00	0.00	0.00	0.72	2601.10
Eastern Messinia 4	0.00	0.00	1.04	192.64	0.00	0.82	0.49	0.18	0.00	0.00	0.00	1.18	2434.83
Eastern Messinia 5	0.00	70.76	0.75	178.78	0.00	1.16	0.00	0.16	0.00	0.00	0.00	0.76	2461.28
Eastern Messinia 6	0.00	68.99	0.76	214.30	0.00	0.82	0.00	0.06	0.00	0.00	0.00	1.00	3030.92

Eastern Messinia 7	0.00	0.00	0.89	191.36	0.00	0.40	0.42	1.01	0.00	0.00	0.00	0.78	2530.52
Eastern Messinia8	0.00	28.47	0.21	117.86	0.00	1.02	0.00	0.21	0.00	0.00	0.00	1.14	2224.59
Eastern Messinia Σ9	0.00	70.49	0.76	202.38	0.00	0.83	0.00	0.00	0.00	0.00	0.00	0.74	2815.10
Eastern Messinia 10	0.00	46.49	0.77	199.19	0.00	1.04	0.00	0.00	0.00	0.00	0.00	0.66	2493.80
Eastern Messinia 11	0.00	51.20	0.90	203.74	0.00	0.99	0.00	0.00	0.00	0.00	0.00	0.55	2698.09
Eastern Messinia 12	0.00	59.49	0.68	189.44	0.00	0.00	0.00	0.00	0.00	0.00	0.00	0.66	2619.19
AVG	***0.00***	***48.14***	***0.74***	***186.13***	***0.00***	***0.77***	***0.08***	***0.14***	***0.00***	***0.00***	***0.00***	***0.79***	**2580.11**
±SD	***0.00***	***25.94***	***0.21***	***27.57***	***0.00***	***0.42***	***0.18***	***0.29***	***0.00***	***0.00***	***0.00***	***0.22***	**206.06**
Lakonia 1	0.00	72.14	0.23	161.07	0.00	1.03	0.00	0.28	0.00	0.00	0.00	1.26	2896.78
Lakonia 2	0.00	0.00	0.11	178.36	0.00	0.26	0.94	1.08	0.00	0.20	0.00	1.53	2720.08
Lakonia 3	0.00	0.00	0.14	154.80	0.00	0.30	0.37	0.57	0.00	0.00	0.00	1.42	2384.60
Lakonia 4	0.00	22.13	1.09	213.17	0.00	0.94	0.00	0.32	0.00	0.00	0.00	1.69	2946.84
Lakonia 5	0.00	15.27	0.21	181.13	0.00	0.79	0.00	0.00	0.00	0.00	0.00	1.09	2674.66
Lakonia 6	0.00	0.00	0.10	180.93	0.00	0.49	0.30	1.00	0.00	0.00	0.00	1.25	2657.75
Lakonia 7	0.00	50.59	0.10	189.49	0.00	1.14	0.64	0.00	0.00	0.00	0.00	0.94	2685.89
Lakonia 8	0.00	70.67	0.26	176.48	0.00	0.03	0.00	0.00	0.00	0.00	0.00	1.03	2945.67
Lakonia 9	0.00	39.99	0.13	177.56	0.00	0.91	0.00	0.41	0.00	0.00	0.00	0.90	2814.85
Lakonia 10	0.00	59.74	0.12	175.09	0.00	1.03	0.00	0.00	0.00	0.00	0.00	0.81	2668.78
Lakonia 11	0.00	0.00	1.59	262.86	0.00	0.37	0.67	0.74	0.00	0.00	0.00	1.87	3677.20
Lakonia 12	0.00	75.88	0.09	211.46	0.00	1.07	0.00	0.00	0.00	0.00	0.00	1.42	2862.61
AVG	***0.00***	***33.87***	***0.35***	***188.53***	***0.00***	***0.70***	***0.24***	***0.37***	***0.00***	***0.02***	***0.00***	***1.27***	**2827.98**
±SD	***0.00***	***31.10***	***0.48***	***28.94***	***0.00***	***0.38***	***0.34***	***0.40***	***0.00***	***0.06***	***0.00***	***0.33***	**310.65**
CV(N=36)	0.00	0.79	0.84	0.23	0.00	1.35	1.83	1.32	0.00	5.08	0.00	0.40	

Each sample was analyzed in triplicate and results were averaged. AVG: average. SD: standard deviation. CV: coefficient of variation; defined as the ratio of standard deviation to the average. It is often expressed as a percentage. N: total number of prickly pear fruit juice samples. TMC: total mineral content (mg/kg).

Supplementary Table 8 (ST8). SPME-GC/MS method optimization/development.

SPME-GC/MS Optimization Procedure	Sample volume (mL)	Extraction Temperature (° C)	Salt addition (NaCl)	Equilibrium time (min)	Sampling time (min)	GC/MS charcteristics*
Trial 1	10	40	NO	20	20	Method 1
Trial 2	10	40	NO	20	20	Method 2
Trial 3	5	40	NO	20	20	Method 1
Trial 4	5	40	NO	20	20	Method 3
Trial 5	5	40	NO	10	20	Method 3
Trial 6	5	40	NO	20	20	Method 3
Trial 7	5	40	NO	30	20	Method 3
Trial 8	5	45	NO	10	20	Method 3
Trial 9	5	45	NO	20	20	Method 3
Trial 10	5	45	NO	30	20	Method 3
Trial 11	5	42	NO	10	20	Method 3
Trial 12	5	42	NO	20	20	Method 3
Trial 13	5	42	NO	30	20	Method 3
Trial 14	5	42	NO	30	10	Method 3
Trial 15	5	42	NO	30	20	Method 3
Trial 16	5	42	NO	30	30	Method 3
Trial 17	5	42	NO	25	20	Method 3
Trial 18	5	42	NO	25	20	Method 3
Trial 19	5	42	NO	25	20	Method 3
Trial 20	5	42	20%	25	20	Method 3
Trial 21	5	42	20%	25	20	Method 3
Trial 22	5	42	30%	25	20	Method 3
Trial 23	5	42	30%	25	20	Method 3
Trial 24	5	42	30%	25	20	Method 3
Trial 25	5	42	30%	25	20	Method 3
Trial 26	5	42	30%	25	20	Method 3
Trial 27	5	42	30%	25	20	Method 3
Trial 28	5	42	30%	25	20	Method 3

Each trial was carried out in duplicate (n = 2). Trials 1, 2, 4 (TIC 1): method 1, 2, 3. Trials 5, 6,7 (TIC 2): equilibrium time and sampling time of 20 min at 40° C water bath. Trials 8, 9, 10 (TIC 3): equilibrium time and sampling time of 20 min at 45° C. Trials 11, 12, 13 (TIC 4): equilibrium time and sampling time of 20 minutes at 42° C water bath. Trials 14, 15, 17 (TIC 5): trial 14, 15 equilibrium time of 30 minutes and 10 and 30 min sampling time respectively; trial 17 equilibrium time of 25 min and sampling time of 20 min at 42 °C. Trials 7, 10, 13 (TIC 6): temperature of 40°C, 45°C, and 42° C (respectively) in water bath; equilibrium time of 30 min and sampling time of 20 min. Trials 13, 17 (TIC 7): equilibrium time of 30 and 25 min, respectively; sampling time of 20 min at 42° C. Trials 20, 22 (TIC 8): equilibrium time of 25 and 20 min, respectively; 20% (w/v) NaCl and 30% (w/v), respectively; water bath at 42° C.

GC/MS characteristics*	Method 1	Method 2	Method 3
Oven temperature	40° C for 2 min, then increased by 10°C to 260 °C for 10 min	40° C for 0 min, then increased by 10°C to 90 °C for 0 min, then increased by 5° C to 220° C for 0 min, and finally increased by 10°C per min to 260 °C for 1 min	40˚C for 0 min, increased to 168 ˚C by 4 ˚C/min (0 min hold) and finally increased with a rate of 10 °C/min to 260 °C (1 min hold):
Split	2:1	2:1	2:1
Air flow	3 mL	3 mL	3 mL
Pressure	8.3755 psi	1.5087 psi	8.3755 psi

Comment/Decision regarding the methodology followed

Method 3 was the most effective method followed in terms of: i) the number of volatiles determined, ii) MS qualification results, iii) the limited furan derivatives identified, and iv) the spectra intensity along with the agreement in volatiles identified during the analysis of replicates. Some typical total ion chromatograms (TIC), pointing out the dominant volatile compounds identified in prickly pear juice such as 1-Hexanol, 2-Hexen-1-ol, and 3-Hexen-1-ol, follow the text sequence. It should be stressed that overlay has been adopted in order to highlight the effectiveness of the final/optimized method followed.

Supplementary Table 9 (ST 9). Mineral content (mg/L) of soil samples according to prickly pear geographical origin.

Soil samples	Al	B	Ba	Be	Ca	Co	Cr	Cu	Fe	K	Li	Mg	Mn	Mo	Na	Ni	P	Sb	Se	Si	Sn	Ti	Tl	V	Zn	TMC
Eastern Messinia	7061.41	46.16	20.33	0.35	54790.36	6.02	10.87	16.25	13670.83	2717.89	22.06	2388.69	281.03	1.79	563.90	17.09	554.08	7.86	0.00	49.27	2.79	0.00	0.00	13.74	0.00	82242.76
Western Messinia	10003.16	57.94	65.93	0.69	3514.77	12.85	76.80	29.45	18393.39	3114.07	17.67	3240.79	1089.49	1.64	578.81	0.00	310.70	0.00	0.00	638.24	1.32	0.00	0.00	29.68	43.64	41221.04
Lakonia	23353.48	75.84	85.78	1.43	42922.12	7.40	40.57	39.63	20724.03	7767.81	43.80	2235.03	385.55	3.94	529.81	24.80	3946.18	36.64	0.00	103.10	145.02	96.29	0.00	37.88	150.02	102606.13

TMC: total mineral content (mg/L). Results reported are the average values of three replicates (n = 3).

Supplementary Table 10. Discriminatory ability of the developed LDA model for the classification of prickly pear juice according to geographical origin based on 7 minerals.

Classification Results [a,b]						
Chemometric techniques		Geographical Origin	Predicted Group Membership			Total Samples (N = 36)
			West Messinia	East Messinia	Lakonia	
Original[a]	Count	Western Messinia	11	1	0	12
		Eastern Messinia	1	10	0	11
		Lakonia	0	0	12	12
		Ungrouped cases	1	0	0	1
	%	Western Messinia	91.7	8.3	.0	100.0
		Eastern Messinia	9.1	90.9	.0	100.0
		Lakonia	.0	.0	100.0	100.0
		Ungrouped cases	100.0	.0	.0	100.0
Cross-validated[b]	Count	Western Messinia	11	1	0	12
		Eastern Messinia	1	9	1	11
		Lakonia	0	2	10	12
	%	Western Messinia	91.7	8.3	.0	100.0
		Eastern Messinia	9.1	81.8	9.1	100.0
		Lakonia	.0	16.7	83.3	100.0

[a.] 94.3% of original grouped cases correctly classified. [b.] Cross validation is done only for those cases in the analysis. In cross validation, each case is classified by the functions derived from all cases other than that case. 85.7% of cross-validated grouped cases correctly classified.

Supplementary Table 11. Discriminatory ability of the developed LDA model for the classification of prickly pear juice according to geographical origin based on 21 volatile compounds.

Classification Results[a,b]

Chemometric techniques		Geographical Origin	Predicted Group Membership West Messinia	East Messinia	Lakonia	Total Samples (N = 36)
Original[a]	Count	Western Messinia	12	0	0	12
		Eastern Messinia	0	12	0	12
		Lakonia	0	0	12	12
	%	Western Messinia	100.0	.0	.0	100.0
		Eastern Messinia	.0	100.0	.0	100.0
		Lakonia	.0	.0	100.0	100.0
Cross-validated[b]	Count	Western Messinia	9	3	0	12
		EasternMessinia	1	11	0	12
		Lakonia	0	0	12	12
	%	Western Messinia	75.0	25.0	.0	100.0
		Eastern Messinia	8.3	91.7	.0	100.0
		Lakonia	.0	.0	100.0	100.0

[a.] 100.0% of original grouped cases correctly classified. [b.] Cross validation is done only for those cases in the analysis. In cross validation, each case is classified by the functions derived from all cases other than that case. 88.9% of cross-validated grouped cases correctly classified.

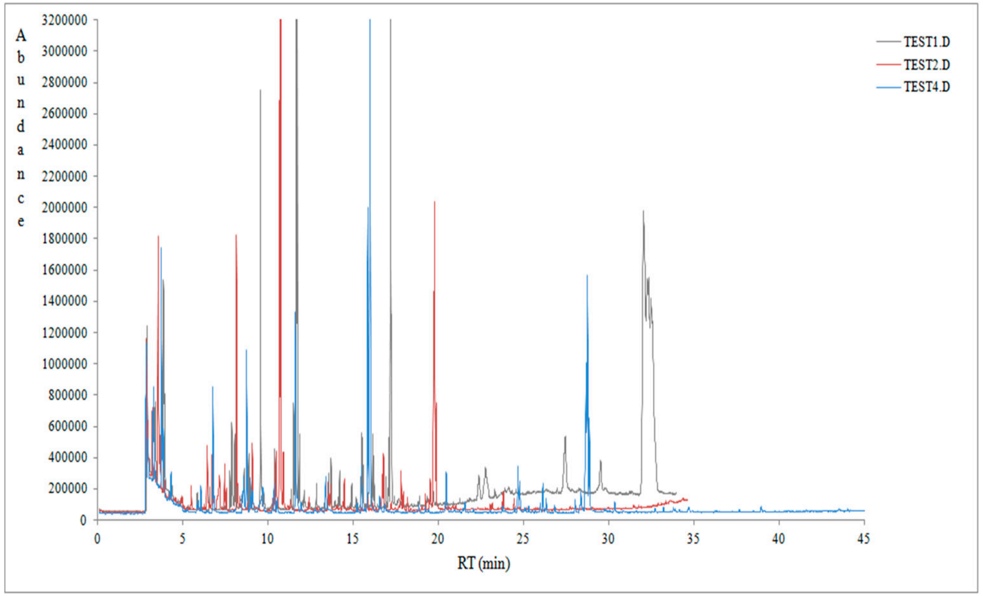

Figure S1. **A** typical gas chromatogram of a prickly pear juice mixture of the 3 regions during method optimization. TEST1, TEST2, and TEST 4 refer to methods 1, 2, 3 (Table S8).

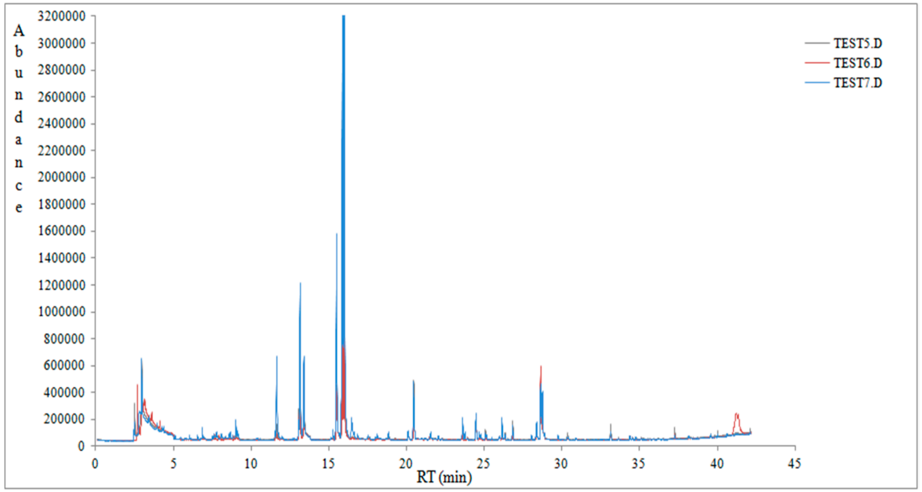

Figure S2. A typical gas chromatogram of a prickly pear juice mixture of the 3 regions during method optimization.TEST5, TEST6, and TEST7 refer to method 3 (Table S8).

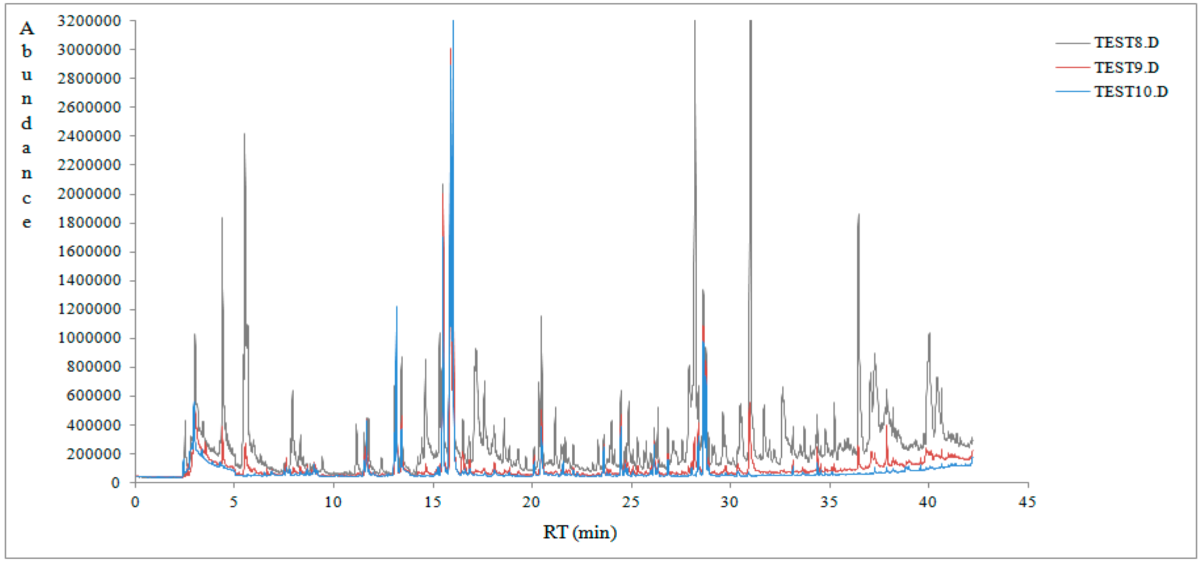

Figure S3. A typical gas chromatogram of a prickly pear juice mixture of the 3 regions during method optimization. TEST8, TEST9, and TEST10 refer to method 3 (Table S8).

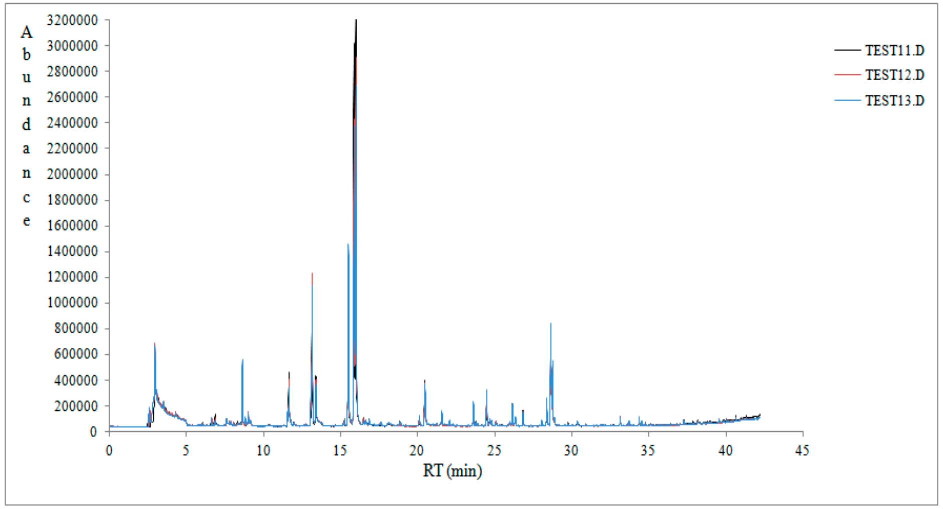

Figure S4. A typical gas chromatogram of a prickly pear juice mixture of the 3 regions during method optimization. TEST11, TEST12, and TEST13 refer to method 3 (Table S8).

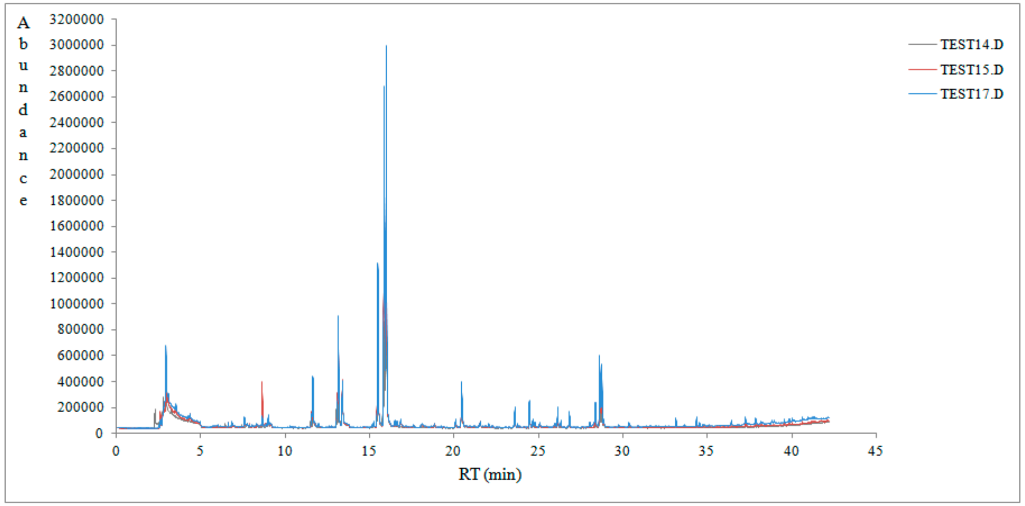

Figure S5. A typical gas chromatogram of a prickly pear juice mixture of the 3 regions during method optimization.TEST14, TEST15, and TEST17 refer to method 3 (Table S8).

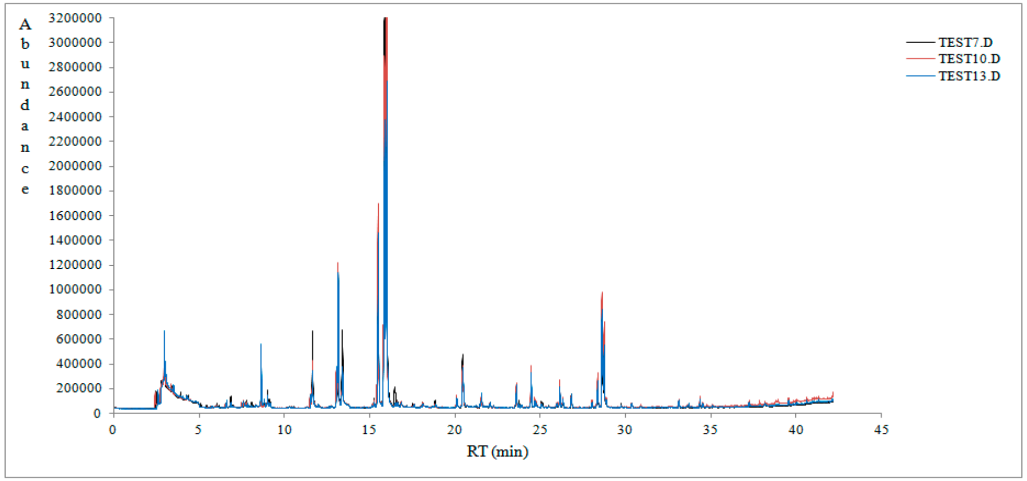

Figure S6. A typical gas chromatogram of a prickly pear juice mixture of the 3 regions during method optimization. TEST7, TEST10, and TEST13 refer to method 3 (Table S8).

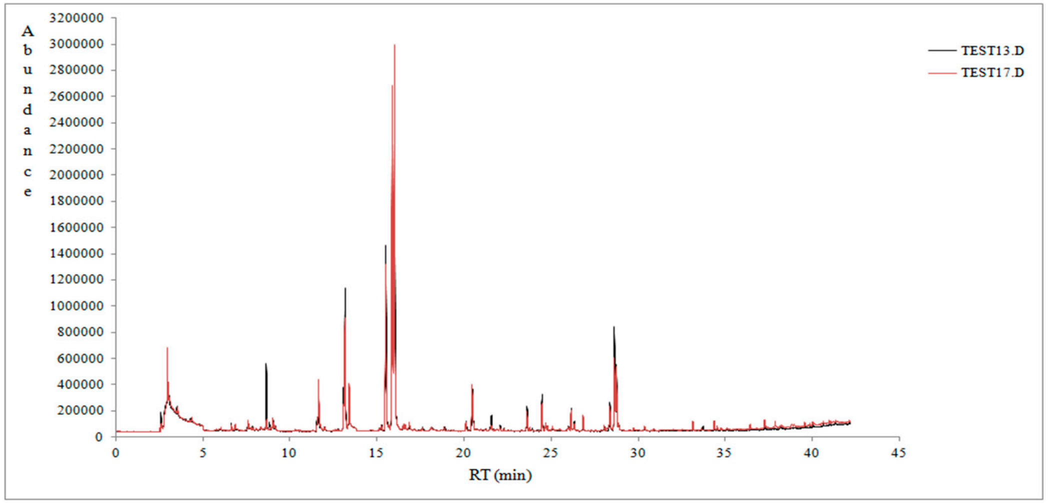

Figure S7. A typical gas chromatogram of a prickly pear juice mixture of the 3 regions during method optimization. TEST13 and TEST17 refer to method 3 (Table S8).

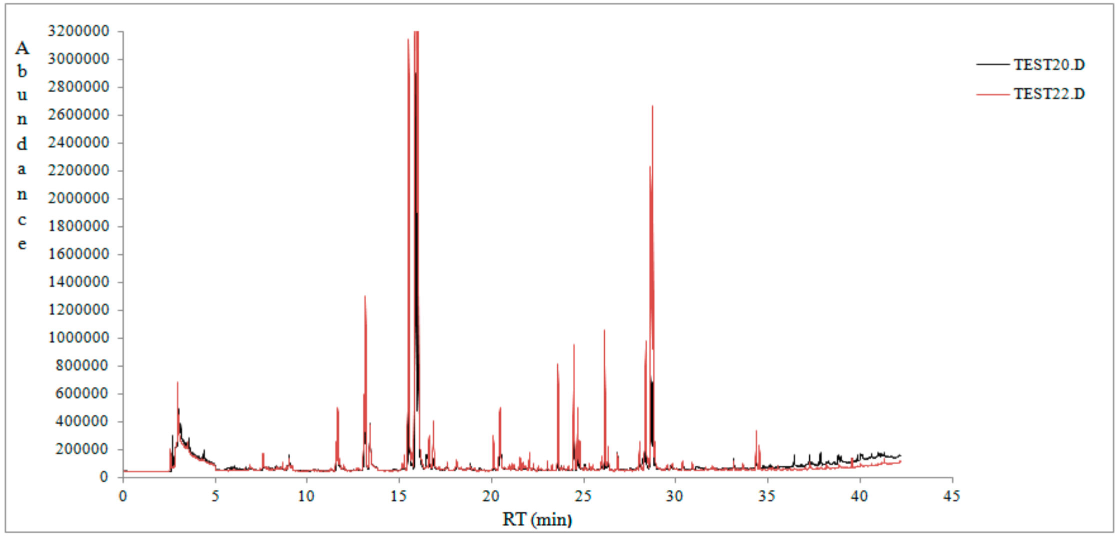

Figure S8. A typical gas chromatogram of a prickly pear juice mixture of the 3 regions during method optimization. TEST20 and TEST22 refer to method 3 (Table S8).
